# Incidence and duration of hospital-initiated opioids, benzodiazepines and antipsychotics: a retrospective cohort study

**DOI:** 10.1007/s11096-026-02152-w

**Published:** 2026-04-30

**Authors:** Judith de Ruijter-van Dalem, Marjo J. A. Janssen, Johanna H. M. Driessen, Carl E. H. Siegert, Alex Marmorale, Daniala L. Weir, Fatma Karapinar-Çarkit

**Affiliations:** 1 Department of Clinical Pharmacy & Toxicology, Maastricht University Medical Center+, P. Debyelaan 25, 6229 HX Maastricht, The Netherlands; 2 Department of Clinical Pharmacy, NUTRIM, Institute of Nutrition and Translational Research in Metabolism, Maastricht University, Maastricht, The Netherlands; 3 Department of Clinical Pharmacy, OLVG Hospital, Amsterdam, The Netherlands; 4 Department of Clinical Pharmacy, CARIM, Cardiovascular Research Institute Maastricht, Maastricht University, Maastricht, The Netherlands; 5 Department of Internal Medicine, OLVG Hospital, Amsterdam, The Netherlands; 6 Epic Systems Corporation, Verona, WI USA; 7 Division of Pharmacoepidemiology and Clinical Pharmacology, Department of Pharmaceutical Sciences, Utrecht Institute of Pharmaceutical Sciences, Utrecht University, Utrecht, The Netherlands

**Keywords:** Analgesics, opioid, Antipsychotic agents, Benzodiazepines, Inappropriate prescribing, Patient discharge, Transitional care

## Abstract

**Introduction:**

Many patients receive temporarily indicated medications, such as opioids, benzodiazepines, and antipsychotics (potentially inappropriate medications (PIMs) of interest) in hospitals. This could result in PIM use thereafter, when medication is continued.

**Aim:**

This study aimed to investigate the incidence and duration of hospital-initiated PIM use.

**Method:**

This retrospective cohort study included patients aged ≥ 18 years who visited a Dutch teaching hospital between January 2019-May 2023 and received a new prescription for an opioid, benzodiazepine, or antipsychotic initiated at discharge or outpatient visit and continued thereafter. Patients were followed for one year using data from the hospital’s information system and community pharmacy dispensing records. The primary outcomes were the incidence of PIMs of interest and duration of use. Secondary outcomes included duration of PIM use across age categories and the proportion of prescriptions classified as actually inappropriate medication based on the documented indications in a subset of patients. Descriptive data analysis was used. Chi-square tests were used to assess differences between age categories and duration of continued use.

**Results:**

Among 6,835 patients with a new prescription of one of the medications, 82.7% were prescribed and dispensed an opioid (n = 5,652, mean age 61.1 years, 57.3% female), 14.7% a benzodiazepine (n = 1,005, mean age 60.7 years, 53.3% female) and 2.6% an antipsychotic (n = 178, mean age 69.2 years, 48.9% female). A significant proportion used the medication > 182 days following the hospital visit (13.4% opioids, 20.9% benzodiazepine, 36.0% of antipsychotics). PIMs were not more frequently discontinued in older adults. Among 156 randomly evaluated prescriptions, a clear justification for continued use was often lacking, mainly for benzodiazepines (83.1%) and opioids (76.0%).

**Conclusion:**

Potentially temporarily indicated medications were often continued following a hospital visit without justification for continued use. These results highlight the importance of carefully assessing the timely discontinuation or specifying a discontinuation date for PIMs.

**Supplementary Information:**

The online version contains supplementary material available at 10.1007/s11096-026-02152-w.

## Impact statements

- The results of this study show that despite more attention to inappropriate use of medications, there is still a large percentage of patients that use hospital-initiated benzodiazepines, opioids and antipsychotics long-term without a valid indication.
- This study highlights the need for targeted interventions to timely address inappropriate medication use.
- Clinicians should carefully evaluate whether these medications can be discontinued or whether a discontinuation date should be specified.

## Introduction

Although hospital visits can provide an opportunity to review and optimize a patient's medication regimen and to reduce inappropriate prescribing, many patients actually receive new temporarily indicated medications when they are hospitalized, such as opioids after surgery, antipsychotics for delirium and benzodiazepines for sleep problems. When such medications are continued following the hospital visit, it can result in potentially inappropriate medication (PIM) prescriptions [1, 2]. While any medication can have an adverse effect, PIMs are those that carry a greater risk of harm than benefit for patients, especially over the age of 65 [3]. Several studies have shown that PIMs are frequently prescribed, especially in older adults, with a prevalence ranging from 24% to almost 44% [3–6], and can result in hospital readmissions, dependency, abuse, harmful events and other adverse effects [7–10].

In the Netherlands, more than 50% of hospitalized patients have at least one PIM listed at the time of admission [11]. However, there is no data on the continuation of hospital-initiated PIMs, particularly opioids, antipsychotics, and benzodiazepines. Studies conducted in other countries have yielded inconsistent findings, with some reporting an increase and others a decrease in PIM prescribing during hospitalization [12, 13]. A recent study also has shown that approximately one in fifteen older adults discharged from the emergency department is prescribed a PIM [14]. However, these studies focus on administrative healthcare data without a review of clinical notes in the patient’s medical record to assess whether there is an indication for the continuation of the medication (e.g. the use of opioids to manage malignant pain). More knowledge is needed about the incidence of actually inappropriate medications, defined as newly prescribed PIMs that are continued after hospital visits without a documented valid indication. However, to date, no previous research has examined the actual inappropriateness of PIMs.

### Aim

The aim of this retrospective cohort study was to investigate the incidence and duration of hospital-initiated PIM use (benzodiazepines, opioids, and antipsychotics). Additionally, prescription indications were evaluated in a random subset of patients.

## Method

### Study design and setting

A retrospective cohort study was conducted in a Dutch teaching hospital (OLVG in Amsterdam) between February 2024 and June 2025. Data from the hospital information system and information obtained via the Nationwide Medication Record System (NMRS) were used to identify all patients who received a new prescription for one of the PIMs of interest (benzodiazepines, opioids, or antipsychotics) initiated at discharge or at an outpatient visit and continued thereafter. Patients were followed for one year to assess incidence and duration of PIM use.

### Usual care

In the Netherlands, medication reconciliation is required at each transition point of care. In the majority of the hospitals, medication reconciliation is performed by pharmacy technicians or by physicians themselves. Medication reconciliation often focuses on obtaining a complete list of medication a patient is using without evaluating the appropriateness of new medication initiated in the hospital. The NMRS, a network enabling healthcare providers to exchange medical data (including medication dispensing data from community pharmacies), is used for medication reconciliation. The network is accessible for healthcare professionals across the country, if the patient provides informed consent to exchange data [15]. At each hospital encounter (including outpatient clinic visits or telephone calls), the NMRS network is queried and the eight-month medication history of the patient is available.

### Study population

Patients aged ≥ 18 years who visited a Dutch teaching hospital between January 2019-May 2023 and received a new prescription for a hospital-initiated opioid, benzodiazepine, or antipsychotic were included and data were abstracted up to one year thereafter.

Only patients with a medication history, i.e. available NMRS information that can be accessed prior to the hospital visit and with medication reconciliation conducted, were included in the study. If available, hospital data covering at least 12 months prior to hospital visit was also used to assess historical medication use.

We excluded all patients who had a recorded dispensing or prescription of any of the medications of interest in the 8 (NMRS) to 12 (hospital system) months before hospital visit. In addition, patients were excluded if they lacked one year of follow-up (i.e. no hospital encounters in which the NMRS was queried, leaving us without information during the one year of follow-up) or if the prescribed PIM was not collected (i.e. no evidence that a pharmacy had actually dispensed the medication to the patient). The latter could occur, for example, if the next healthcare professional discontinued the medication shortly after the hospital visit. Finally, patients that died in the hospital, transferred to another hospital, or objected to the use of their information for research purposes (as recorded in the hospital system) were also excluded.

For assessing actually inappropriate medications, 50 patients from the overall study cohort were selected randomly for each medication group (50 users of benzodiazepines, 50 users of opioids and 50 users of antipsychotics). As this was an exploratory investigation, no formal sample size calculation was performed, and a pragmatic sample of patients was included. For each medication group, patients were randomly selected with SPSS (simple random sampling without replacement) with sampling performed independently within each medication group based on duration of use: 10 patients with use lasting 30 to 182 days, 20 patients with use between 183 and 365 days, and 20 patients with use exceeding 365 days. This ensured inclusion of both short-term and long-term PIM users, as clinical appropriateness of benzodiazepines, opioids and antipsychotics could be time-dependent, to assess whether continued use of PIMs was actually inappropriate. Patients could use more than one PIM within the same medication group (e.g., patients using two different benzodiazepines); in such cases, the indication was evaluated for all prescriptions of interest.

### Study measures

Extracted data included information on the use of the medications of interest (e.g. start date, end date, dose and amount) after hospital visit and clinical characteristics. Use of a benzodiazepine, opioid or antipsychotic was identified from the electronic patient health record system using Anatomical Therapeutical Classification System codes (ATC-codes). To determine the duration of medication use after hospital visit, we used information obtained via the NMRS. Clinical characteristics of interest were collected from the electronic patient health record system and included: age, sex, department, discharge destination (home, nursing home or other) and length of hospital stay.

To evaluate whether the continued use of a PIM was justified, clinical notes from patients’ records were reviewed for indications of the hospital-initiated medication. The assessment applied predefined criteria, initially based on the STOPP (Screening Tool of Older Persons' potentially inappropriate Prescriptions) criteria and Beers criteria (Online Resource 1) [16, 17] and was further refined by an expert panel consisting of a hospital pharmacist–clinical pharmacologist-Board Certified Geriatric Pharmacist and an internal medicine physician, to determine whether a PIM could be considered appropriate or not. For example, a specific visual analogue scale (VAS) score was added to define mild or moderately severe non-malignant (chronic) pain. The expert panel used the refined criteria to determine whether continuation of a PIM was clinically justified. When no valid indication for continuing a hospital-initiated PIM was documented in the patient’s record, the medication was classified as actually inappropriate. In cases where there was uncertainty or disagreement, the cases were discussed to reach consensus.

### Outcomes

Primary outcomes of this study were the incidence and duration of hospital-initiated PIM prescriptions for each of the medication classes of interest. Secondary outcomes included identifying the individual medications most frequently prescribed within the PIM category, assessing whether incidence varied by age group and assessing temporal trends influences (by assessing results for each year separately). Additionally, the incidence of actually inappropriate medication prescriptions in a random subset of patients was evaluated.

### Statistical analysis

All data were recorded in a Microsoft Excel spreadsheet (Microsoft Corporation, Redmond, WA, USA). Descriptive clinical characteristics of the cohort were reported for new PIM use of benzodiazepines, opioids and antipsychotics. Descriptive statistics were used to calculate PIM incidence rates and were stratified according to the duration of use and age. Duration of use was classified into < 30, 30–182, 183–365 and > 365 days and age was classified into < 65 and 65 + years for a duration of use of < 30, 30–182, > 182 days. Chi-square tests were used to post-hoc assess differences in the continuation of PIM prescriptions between age categories and duration of continued use, analysed separately for each medication group. A *p*-value lower than 0.05 was considered statistically significant. Moreover, the duration of PIM use was assessed separately for each year within the study period to examine whether temporal trends influenced the results. The percentage of actually inappropriate medications was calculated relative to the total number of PIMs reviewed.

All analyses, including selecting the random sample, were performed using IBM SPSS Statistics version 28.0 (IBM Corporation, Armonk, NY, USA).

### Ethics approval

The Medical Ethics Committee of the OLVG hospital (reference ID: WO.24.006) waived ethical approval as the study fell outside the scope of the Dutch Medical Research Involving Human Subjects Act (WMO).

## Results

### Patient and medication characteristics

Table 1 summarizes characteristics of 6,835 new PIM users: 14.7% (n = 1,005) benzodiazepine, 82.7% (n = 5,652) opioid, and 2.6% (n = 178) antipsychotic users. A small number of patients (3.3%, n = 228) used more than one PIM, most commonly a combination of a benzodiazepine and an opioid (78.1%, n = 178, data not shown). Benzodiazepine users had a mean age of 60.7 years and 53.3% (n = 536) were female. Most patients visited the internal medicine (16.4%, n = 165), cardiology (14.9%, n = 150), or general surgery (12.4%, n = 125) department. Oxazepam (41.3%, n = 415), temazepam (31.5%, n = 317) and lorazepam (10.0%, n = 100) were the most frequently prescribed benzodiazepines. Users of opioids were 61.1 years of age and 57.3% (n = 3,238) were female. They commonly visited the general surgery (37.9%, n = 2,141) or orthopedics (21.7%, n = 1,227) department. Oxycodone (80.8%, n = 4569) and tramadol (15.6%, n = 881) were the most commonly prescribed opioids. Antipsychotic users were older (69.2 years), less often female (48.9%, n = 87), and mainly visited the internal medicine (25.8%, n = 46) or psychiatry (12.4%, n = 22) department. Quetiapine (39.3%, n = 70), haloperidol (34.8%, n = 62) and olanzapine (14.0%, n = 25) were the most frequent newly prescribed antipsychotics continued following hospital visit.

**Table 1:** Baseline characteristics of patients with a potentially inappropriate medication

	Benzodiazepines		Opioids		Antipsychotics	
Characteristics	(n = 1,005)		(n = 5,652)		(n = 178)	
Female	536	(53.3)	3,238	(57.3)	87	(48.9)
Age, years (mean, SD)	60.7	(17.4)	61.1	(17.8)	69.2	(16.9)
18–64	516	(51.3)	2,869	(50.8)	61	(34.3)
65–74	238	(23.7)	1,279	(22.6)	35	(19.7)
75–84	198	(19.7)	1,116	(19.7)	48	(27.0)
85 +	53	(5.3)	388	(6.9)	34	(19.1)
*Department of hospital visit*						
Cardiology	150	(14.9)	155	(2.7)	19	(10.7)
Gastrointestinal liver department	68	(6.8)	229	(4.1)	< 5	
Geriatric department	22	(2.2)	60	(1.1)	14	(7.9)
Gynaecology	67	(6.7)	221	(3.9)	9	(5.1)
Internal medicine	165	(16.4)	271	(4.8)	46	(25.8)
Neurology	104	(10.3)	127	(2.2)	16	(9.0)
Orthopedics	20	(2.0)	1,227	(21.7)	3	(1.7)
Pulmonology	103	(10.2)	182	(3.2)	12	(6.7)
Surgery (general)	125	(12.4)	2,141	(37.9)	17	(9.6)
Surgery (other)	56	(5.6)	546	(9.6)	< 5	
Psychiatry	14	(1.4)	< 5		22	(12.4)
Urology	31	(3.1)	190	(3.4)	< 5	
Other	80	(8.0)	302	(5.4)	14	(7.9)
*Discharge destination*						
Home	886	(88.2)	5,074	(89.8)	132	(74.2)
Nursing home	17	(1.7)	88	(1.6)	20	(11.2)
Other	42	(4.2)	204	(3.6)	17	(9.6)
Not applicable (day admission)	60	(6.0)	286	(5.1)	9	(5.1)

Data are presented as number (%) of individuals, unless stated otherwise

Abbreviation: *n* Number, *SD* Standard deviation

### Duration of use

**Fig. 1:**
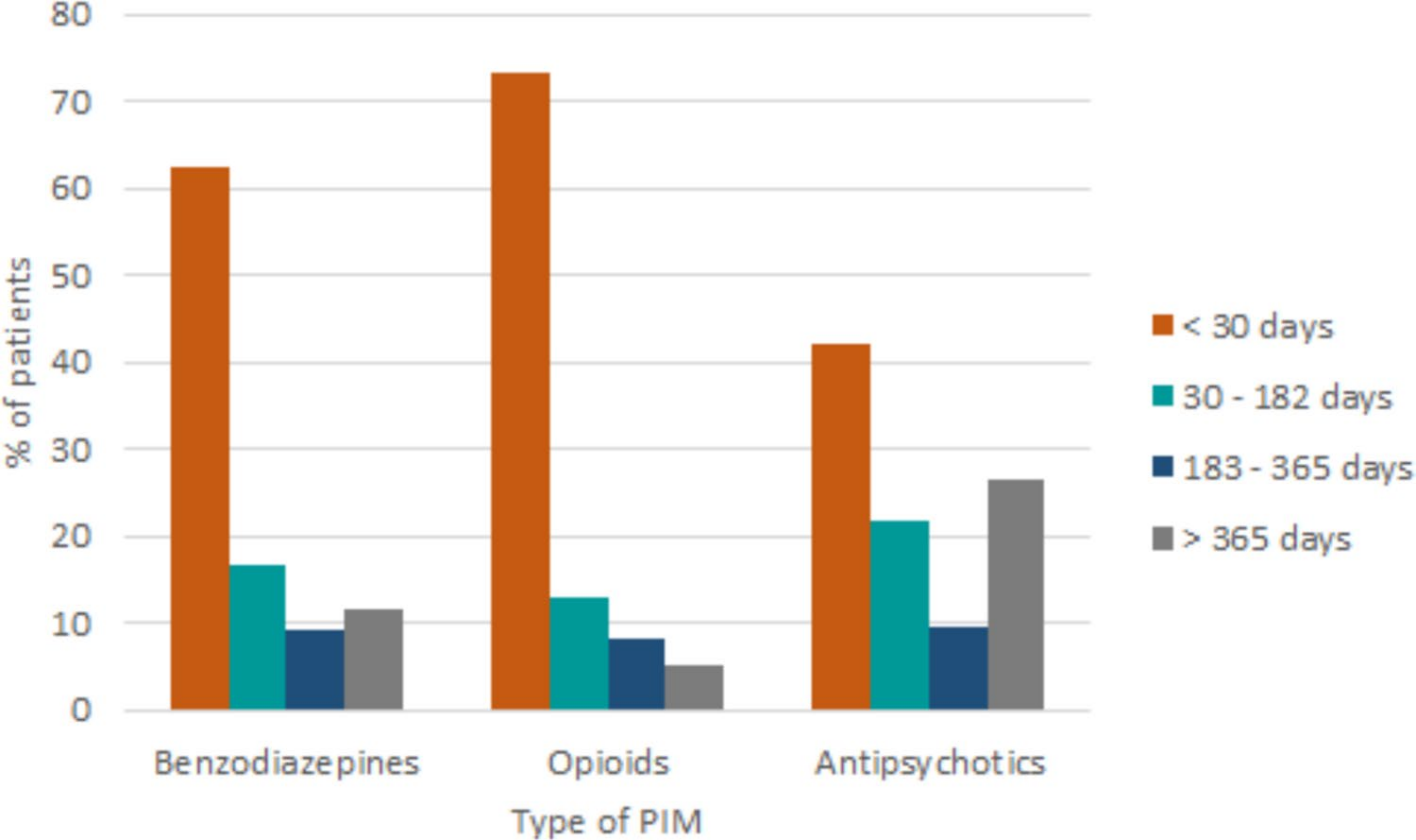
Duration of potentially inappropriate medication (PIM) use after hospital visit

Figure 1 shows that most of the patients were short-term users of the PIMs. 62.5% (n = 628) of the benzodiazepine users, 73.4% (n = 4,151) of the opioid users and 42.1% (n = 75) of the antipsychotic users had a duration of use of less than 30 days. However, 20.9% (n = 210) of benzodiazepine users, 13.4% (n = 760) of opioid users, and 36.0% (n = 64) of antipsychotic users continued their medication for longer than six months. Among patients who used these medications for more than six months, 44% (n = 455) continued them for over one year. These results were not influenced by temporal trends and were consistent within each year (data not shown). When results were stratified by age (< 65 years and 65 +), no lower incidence of PIM use was observed among patients aged ≥ 65 years compared with younger patients for benzodiazepine and antipsychotic users. Among opioid users, prolonged continuation was significantly more common in patients aged ≥ 65 years than in those aged < 65 years, although differences were small (Online Resource 2).

### Actually inappropriate medication

A total of 156 prescriptions were evaluated in 147 patients to evaluate if PIMs were actually inappropriate medications, comprising benzodiazepines (n = 59), opioids (n = 50), and antipsychotics (n = 47) (Table 2). We only included 47 users of antipsychotics as there were only 17 users of antipsychotics with a duration of use of 183–365 days in the cohort. Consensus was reached for all evaluated cases. Among benzodiazepine prescriptions, 83.1% (n = 49) were classified as inappropriate. Inappropriate use was most frequently related to prescriptions for insomnia (28 of 29 benzodiazepine prescriptions) and anxiety (11 of 14 benzodiazepine prescriptions) as short-term use is advised in guidelines. However, one prescription for insomnia was classified as appropriate as it was linked to a diagnosis of non-small cell lung carcinoma. Long-term use in three patients receiving benzodiazepines for anxiety was also considered appropriate as they received multiple prescriptions over an extended period for anxiety related to physical examinations, such as MRI scans.

**Table 2:** Incidence of appropriate and inappropriate prescriptions in a subset of patients

Medication use stratified by indication	Number of prescriptions n	Appropriate prescriptions n (%)	Inappropriate prescriptions n (%)
*Benzodiazepines*^a^	59	10 (16.9)	49 (83.1)
Insomnia	29	1 (1.7)	28 (47.5)
Anxiety	14	3 (5.1)	11 (18.6)
Other	16	6 (10.2)	10 (16.9)
*Opioids*	50	12 (24.0)	38 (76.0)
Pain post-surgery	20	0 (0.0)	20 (40.0)
Oncological pain	6	6 (12.0)	0 (0.0)
Back pain/joint pain	5	0 (0.0)	5 (10.0)
Other	19	6 (12.0)	13 (26.0)
*Antipsychotics*^b^	47	28 (59.6)	19 (40.4)
Insomnia	11	0 (0.0)	11 (23.4)
Depression	8	8 (17.0)	0 (0.0)
Psychotic episode	7	7 (14.9)	0 (0.0)
Other	21	13 (27.7)	8 (17.0)

^a^Some patients used more than 1 benzodiazepine

^b^There were only 17 users of antipsychotics with a duration of use of 183–365 days in the cohort

For opioids, 76.0% (n = 38) of all prescriptions were classified as inappropriate. Inappropriate use was most often linked to prescriptions for postsurgical pain (20 of 20 opioid prescriptions) and for back or joint pain (5 of 5 opioid prescriptions) as current guidelines advise using opioids only short-term or to avoid them. Prescriptions for oncological pain were considered appropriate.

For antipsychotics, inappropriate prescribing was less prevalent compared with the other medication classes. Overall, 40.4% (n = 19) of prescriptions were inappropriate. Inappropriate use occurred most often in patients using an antipsychotic for insomnia (11 of 11 antipsychotic prescriptions) as off-label treatment. In contrast, all prescriptions for complex depression, e.g. with psychotic features (8 of 8 antipsychotic prescriptions) and all prescriptions for psychotic episodes (7 of 7 antipsychotic prescriptions) were considered appropriate as this is guideline recommended treatment. Only three prescriptions had delirium as the indication. All were considered inappropriate for long-term continuation as delirium is expected to resolve within a few weeks.

## Discussion

The results of this retrospective cohort study provide important insights into the persistence of hospital-initiated PIMs and continued thereafter. The findings show that a substantial proportion of patients who were newly prescribed benzodiazepines, opioids or antipsychotics continued to use these medications well beyond the acute period. More than one in five benzodiazepine users, over one in ten opioid users, and more than one in three antipsychotic users continued their medication for longer than six months. After evaluation of indications, actually inappropriate prescribing was common, with particularly high percentages observed for benzodiazepines (83.1%) and opioids (76.0%). These findings highlight the challenges in managing medications across transitions in care.

The findings of this study are consistent with previous research investigating the persistent use of PIMs. The high prevalence of prolonged benzodiazepine use in our cohort (20.9% with use for more than six months) is in line with findings from previous studies. These have shown that, despite guidelines recommending short-term use only, benzodiazepines are often continued for extended periods after discharge or in other settings, including in older adults [18, 19]. Gerlach et al. [19] reported that 26.4% of patients, with a mean age of 78 years, still used a benzodiazepine one year following the index prescription. We also found that one in ten patients used a hospital-initiated opioid for more than six months. However, previous studies have reported varying rates of continued opioid use, with estimates of long-term use (≥ three to six months) ranging from 0.5% to over 15% depending on patient population, used definitions and setting [20–22]. Our study also found that 36.0% of patients with a new antipsychotic prescription continued use beyond six months. Studies show that continuation of antipsychotics initiated in hospital settings is common [23–25]. Moreover, the study of Madey et al. [26] showed similar results with 31.7% continued antipsychotic therapy at discharge. Of these patients, 80 percent were still using it at 30 days, 55 percent at 90 days, and 40 percent at 180 days post-discharge. Interestingly, we found no evidence that temporal trends influenced our results as the results were similar over the years. Given the growing attention to inappropriate prescribing in recent years, we expected a decline over time, but this was not observed. The study period may have been too short to detect this, however, the findings also indicate that inappropriate prescribing remains a persistent problem. Another noteworthy finding was that we did not observe lower (long-term) PIM use in older adults. Moreover, among opioid users, prolonged continuation was more common in patients aged ≥ 65 years than in younger patients, although overall difference between groups was small. We had expected physicians to be more aware of long-term risks in older adults and therefore more timely discontinuation of these medications. Our findings do not support this expectation. This highlights a need for improved deprescribing practices in this vulnerable population.

Previous studies often focused on PIM use, while it is more important to assess actually inappropriate medication use. While there is some literature indicating large numbers of actual inappropriate use of antipsychotic medication use in long-term care facilities [27, 28] and benzodiazepines in primary care [29], we are the first (to the best of our knowledge) to investigate the actual inappropriateness of benzodiazepines, opioids and antipsychotics newly started in the hospital and continued thereafter. Benzodiazepines and opioids are associated with dependence, cognitive impairment, falls/higher fracture risk, overdose and mortality [30–36], while antipsychotics, especially when used long-term in older adults, carry increased risks of cerebrovascular events and mortality [37–39]. Despite these risks, our analysis of the review of clinical notes indicates that most PIM prescriptions were indeed inappropriate for the majority of patients, especially for benzodiazepines and opioids, where long-term use should be discouraged and alternatives are preferred. Antipsychotics were less frequently prescribed inappropriately, these patients were more likely to present with a more complex clinical profile.

The results align with concerns regarding the overuse and inappropriate continuation of medications that carry high risks when used chronically. Several factors may contribute to the continued use of inappropriate medication, including hospitalization itself [40]. First, insufficient transfer of medication information between hospitals and primary care may occur [41]. Moreover, discharge summaries, which may contain additional information about intended use after discharge, are often delayed [41]. In the absence of explicit discontinuation plans or stop dates on the prescription, health care professionals in primary care may assume that continuation of the medication is needed. Additionally, general practitioners may not have the time to carefully evaluate repeat prescriptions which results in long-term continuation. It would be interesting to further investigate the underlying causes of continuation of these medications. However, to prevent dependency in patients, we strongly recommend handling this problem at the source, e.g. at the hospital visit. Integrating deprescribing strategies, e.g. with mandatory discontinuation date documentation, could be useful.

### Strengths and limitations

This study has several notable strengths and limitations. A key strength is the inclusion of all adult patients rather than restricting the cohort to those aged 65 and older, which increases the generalizability of our findings and shows that age does not impact PIM use. Additionally, this is the first study to distinguish between PIMs and actually inappropriate medications, allowing for more precise information about the use of inappropriate medication. However, due to the retrospective design of the study, we used healthcare data collected for clinical purposes rather than research, which may introduce biases and affect data completeness. We also lacked information on patient medication adherence, potentially leading to misclassification of PIM use. Moreover, we lost many patients who did not have follow-up data that could be obtained via the NMRS, either because they did not provide informed consent for the use of the NMRS in a pharmacy or because they were followed-up by other healthcare providers (e.g. primary care, nursing home or rehabilitation care). This could introduce selection bias as patients followed-up by the hospital may be more complex.

## Conclusion

Potentially temporarily indicated medications were often continued following a hospital visit without justification for continued use. Therefore, this study highlights the need for targeted interventions to address PIM use, both for adults and older patients. Recognizing PIMs is the first step. Subsequently integrating deprescribing strategies may help reduce the burden of inappropriate long-term medication use. We strongly emphasize the need for careful evaluation of timely discontinuing these medications or specifying a discontinuation date to inform patients and next healthcare providers.

## Supplementary Information

Below is the link to the electronic supplementary material.

Supplementary file1 (DOCX 17 KB)

Supplementary file2 (DOCX 19 KB)

## Data Availability

The datasets generated during and/or analysed during the current study are available from the corresponding author on reasonable request.
