# Supplementary material for: Incidence and duration of hospital-initiated opioids, benzodiazepines and antipsychotics: a retrospective cohort study
Source: Int J Clin Pharm. 2026 Apr 30;48(4):1694–702. doi: 10.1007/s11096-026-02152-w (PMC13368891; doi:10.1007/s11096-026-02152-w)
Supplement: Supplementary file 1 — Supplementary file1 (DOCX 17 KB) [file 11096_2026_2152_MOESM1_ESM.docx]

**Incidence and duration of potentially inappropriate continuation of in-hospital-initiated opioids, benzodiazepines and antipsychotics: a retrospective cohort study**

Judith de Ruijter – van Dalem PharmD, PhD a,b; Marjo JA Janssen PharmD, PhD c; Johanna HM Driessen PhD a,d; Carl EH Siegert MD e; Alex Marmorale BSc f; Daniala L Weir PhD g; Fatma Karapinar- Çarkit PharmD, PhD a,d

a Department of Clinical Pharmacy & Toxicology, Maastricht University Medical Center+, Maastricht, the Netherlands

b Department of Clinical Pharmacy, NUTRIM, Institute of Nutrition and Translational Research in Metabolism, Maastricht University, Maastricht, the Netherlands

c Department of Clinical Pharmacy, OLVG Hospital, Amsterdam, the Netherlands

d Department of Clinical Pharmacy, CARIM, Cardiovascular Research Institute Maastricht, Maastricht University, Maastricht, the Netherlands

e Department of Internal Medicine, OLVG Hospital, Amsterdam, the Netherlands

f Epic Systems Corporation, Verona, Wisconsin, United States

g Division of Pharmacoepidemiology and Clinical Pharmacology, Department of Pharmaceutical Sciences, Utrecht University, Utrecht, the Netherlands; Utrecht Institute of Pharmaceutical Sciences, Department of Pharmaceutical Sciences, Utrecht University, Utrecht, the Netherlands

Correspondence to

Fatma Karapinar-Çarkıt, PharmD, PhD, e-mail: [f.karapinar@mumc.nl](mailto:f.karapinar@mumc.nl)

| **Online resource 1.** Criteria to determine if a PIM is an AIM ^38,39^ | | |
| --- | --- | --- |
| ***Medication*** | ***Appropriate*** | ***Inappropriate (unless reason for long term use is explicitly specified in the patient records)*** |
| Antipsychotics | Prescribed by a psychiatrist for a potential long-term indication (e.g. schizophrenia, bipolar disorder, Parkinson disease, psychosis, adjunctive treatment of major depressive disorder). | Prescribed by any other specialism than psychiatry. |
|  |  | Indication delirium or sleeping disorder. |
|  |  | In patients with parkinsonism (with the exception of clozapine and quetiapine). |
|  |  | In patients with problem behavior in dementia, unless symptoms are severe or in the acute phase and non-medicinal measures have no effect. |
|  |  | Antipsychotic with moderate anticholinergic effect (chlorpromazine, flupentixol, fluphenazine, zuclopenthixol) in case of prostatism or history of urinary retention. |
|  |  | Prescribed in patients with a high risk of falling (e.g. history of falls, higher age (65+), neuropathy, vision problems, alcohol abuse^38^). |
| Benzodiazepines | Prescribed for the indications: seizure disorders, rapid eye movement, rapid eye movement (REM) sleep behavior disorder, withdrawal symptoms related to addictions, severe generalized anxiety disorder | Prescribed for any other indication (e.g. sleeping problems in the hospital). |
|  |  | Prescribed in patients with a high risk of falling (e.g. history of falls, higher age (65+), neuropathy, vision problems, alcohol abuse^40^). |
| Opioids | Prescribed for the indication malignant pain | Indication mild or moderately severe non-malignant (chronic) pain (VAS score lower than 7.5^41^). |
|  | Prescribed for short-term for the indication severe acute pain (e.g. after surgery) if paracetamol or a NSAID are not effective or contra-indicated | Prescribed in patients with a high risk of falling (e.g. history of falls, higher age (65+), neuropathy, vision problems, alcohol abuse^40^). |
|  |  | Long-term prescriptions of short-acting opioid. |

38. O'Mahony D, Cherubini A, Guiteras AR, Denkinger M, Beuscart JB, Onder G, et al. STOPP/START criteria for potentially inappropriate prescribing in older people: version 3. Eur Geriatr Med. 2023;14(4):625-32.

39. By the American Geriatrics Society Beers Criteria Update Expert P. American Geriatrics Society 2023 updated AGS Beers Criteria(R) for potentially inappropriate medication use in older adults. J Am Geriatr Soc. 2023;71(7):2052-81.

40. CDC. Facts About Falls, Older Adult Fall Prevention. May 9, 2024. https://www.cdc.gov/falls/data-research/facts-stats/index.html

41. Boonstra, Anne M.a,*; Schiphorst Preuper, Henrica R.b; Balk, Gerlof A.a; Stewart, Roy E.c. Cut-off points for mild, moderate, and severe pain on the visual analogue scale for pain in patients with chronic musculoskeletal pain. Pain 155(12):p 2545-2550, December 2014. | DOI: 10.1016/j.pain.2014.09.014
