# Supplementary material for: Incidence and duration of hospital-initiated opioids, benzodiazepines and antipsychotics: a retrospective cohort study
Source: Int J Clin Pharm. 2026 Apr 30;48(4):1694–702. doi: 10.1007/s11096-026-02152-w (PMC13368891; doi:10.1007/s11096-026-02152-w)
Supplement: Supplementary file 2 — Supplementary file2 (DOCX 19 KB) [file 11096_2026_2152_MOESM2_ESM.docx]

**Incidence and duration of potentially inappropriate continuation of in-hospital-initiated opioids, benzodiazepines and antipsychotics: a retrospective cohort study**

Judith de Ruijter – van Dalem PharmD, PhD a,b; Marjo JA Janssen PharmD, PhD c; Johanna HM Driessen PhD a,d; Carl EH Siegert MD e; Alex Marmorale BSc f; Daniala L Weir PhD g; Fatma Karapinar- Çarkit PharmD, PhD a,d

a Department of Clinical Pharmacy & Toxicology, Maastricht University Medical Center+, Maastricht, the Netherlands

b Department of Clinical Pharmacy, NUTRIM, Institute of Nutrition and Translational Research in Metabolism, Maastricht University, Maastricht, the Netherlands

c Department of Clinical Pharmacy, OLVG Hospital, Amsterdam, the Netherlands

d Department of Clinical Pharmacy, CARIM, Cardiovascular Research Institute Maastricht, Maastricht University, Maastricht, the Netherlands

e Department of Internal Medicine, OLVG Hospital, Amsterdam, the Netherlands

f Epic Systems Corporation, Verona, Wisconsin, United States

g Division of Pharmacoepidemiology and Clinical Pharmacology, Department of Pharmaceutical Sciences, Utrecht University, Utrecht, the Netherlands; Utrecht Institute of Pharmaceutical Sciences, Department of Pharmaceutical Sciences, Utrecht University, Utrecht, the Netherlands

Correspondence to: Fatma Karapinar-Çarkıt, PharmD, PhD, e-mail: [f.karapinar@mumc.nl](mailto:f.karapinar@mumc.nl)

| **Online Resource 2.** Duration of potentially inappropriate medication use stratified by age | | | | | | | | | |
| --- | --- | --- | --- | --- | --- | --- | --- | --- | --- |
|  |  | **Benzodiazepines** | | | **Opioids** | | | **Antipsychotics** | |
| Duration of use | | **(n = 1,005)** | | | **(n = 5,652)** | | | **(n = 178)** | |
| **Age < 65 years** | | **516** | **(51.3)** | **2,869** | | **(50.8)** | **61** | | **(34.3)** |
|  | <30 days^a^ | 317 | (61.4) | 2,165 | | (75.5) | 23 | | (37.7) |
|  | 30-182 days^a^ | 99 | (19.2) | 326 | | (11.4) | 15 | | (24.6) |
|  | > 182 days^a^ | 100 | (19.4) | 378 | | (13.1) | 23 | | (37.7) |
|  |  |  |  |  | |  |  | |  |
| **Age 65+ years** | | **489** | **(48.7)** | **2,783** | | **(49.2)** | **117** | | **(65.7)** |
|  | <30 days^b^ | 311 | (63.5) | 1,986 | | (71.4) | 52 | | (44.4) |
|  | 30-182 days^b^ | 68 | (13.9) | 415 | | (14.9) | 24 | | (20.5) |
|  | > 182 days^b^ | 110 | (22.5) | 382 | | (13.7) | 41 | | (35.0) |
| p-value^c^ | | p = 0.062 | | p < 0.001 | | | p = 0.665 | | |
| Data are presented as number (%) of individuals  Abbreviations: n, number.  ^a^ Percentage calculated as the proportion of the total number of patients aged <65 years  ^b^ Percentage calculated as the proportion of the total number of patients aged 65+ years  ^c^ p-values for comparisons between age categories per medication group and duration of continued use. | | | | | | | | | |
